# Immature remains and the first partial skeleton of a juvenile *Homo naledi*, a late Middle Pleistocene hominin from South Africa

**DOI:** 10.1371/journal.pone.0230440

**Published:** 2020-04-01

**Authors:** Debra R. Bolter, Marina C. Elliott, John Hawks, Lee R. Berger

**Affiliations:** 1 Evolutionary Studies Institute and Centre for Excellence in PalaeoSciences, University of the Witwatersrand, Johannesburg, South Africa; 2 Department of Anthropology, Modesto Junior College, Modesto, California, United States of America; 3 Department of Anthropology, University of Wisconsin-Madison, Madison, Wisconsin, United States of America; Max Planck Institute for the Science of Human History, GERMANY

## Abstract

Immature remains are critical for understanding maturational processes in hominin species as well as for interpreting changes in ontogenetic development in hominin evolution. The study of these subjects is hindered by the fact that associated juvenile remains are extremely rare in the hominin fossil record. Here we describe an assemblage of immature remains of *Homo naledi* recovered from the 2013–2014 excavation season. From this assemblage, we attribute 16 postcranial elements and a partial mandible with some dentition to a single juvenile *Homo naledi* individual. The find includes postcranial elements never before discovered as immature elements in the sub-equatorial early hominin fossil record, and contributes new data to the field of hominin ontogeny.

## Introduction

Our knowledge of maturational processes and life history stages in human evolution is hindered by how extremely rare immature remains are in the hominin fossil record. Further complicating our understanding is that hominin fossil remains rarely combine teeth, cranial and associated postcranial bones, and only a handful of such partial skeletons represent immature individuals. Yet such remains provide important evidence about the maturation of extinct hominins. Apart from recent modern humans and Neanderthals (e.g., [1]), only three hominin species are represented by immature partial skeletons at this time: *Australopithecus afarensis* [2, 3], *Australopithecus sediba* [4,5] and *Homo erectus* [6–9]. Augmenting this limited dataset is of great significance in trying to understand the evolution of human growth and development.

The immature individuals from the Dinaledi and Lesedi assemblages of *Homo naledi* represent individuals of many juvenile and adult life stages, presenting a unique opportunity to identify and document in particular a range of immature individuals of an extinct species, and contribute data to the study of comparative hominin ontogeny [10–13]. The Lesedi Chamber sample includes an associated partial skeleton of an adult individual, a jaw fragment from a second adult, and the remains of a very young juvenile [12]. The Dinaledi Chamber sample is much richer in fossil material, with the remains of a minimum of 15 individuals, ranging in age from neonates to older adults and dating to between 335,000 and 226,000 years ago [10,11,13,14].

The geology and taphonomy of the Dinaledi Chamber material has been described previously by Dirks et al. [11]. To sum up, the Dinaledi Chamber is an approximately 3 x 5 m chamber located within an interconnected network of cave passages and spaces now designated as the Dinaledi Subsystem [15]. The Subsystem itself is connected to the larger Rising Star Cave System by a narrow, 12 m high vertical passage known as the Chute [11]. Like most other hominin fossil sites in the area, the cave system formed within the stromatolite-rich dolomite of the Monte Christo Formation [16]. The fossils are not encased in hard breccia, but are recovered from largely unconsolidated mudstone sediments that are distinct from those outside the subsystem, suggesting an autochthonous derivation [11]. The presence of some articulated and semi-articulated remains in the Dinaledi deposit, as well as small elements that normally disarticulate and disperse quickly, suggest limited post-mortem disruption and minimal winnowing. Despite this, the high density of material and the lack of stratigraphy within the sediments make the sequence of deposition and disarticulation challenging to determine.

**Fig 1:**
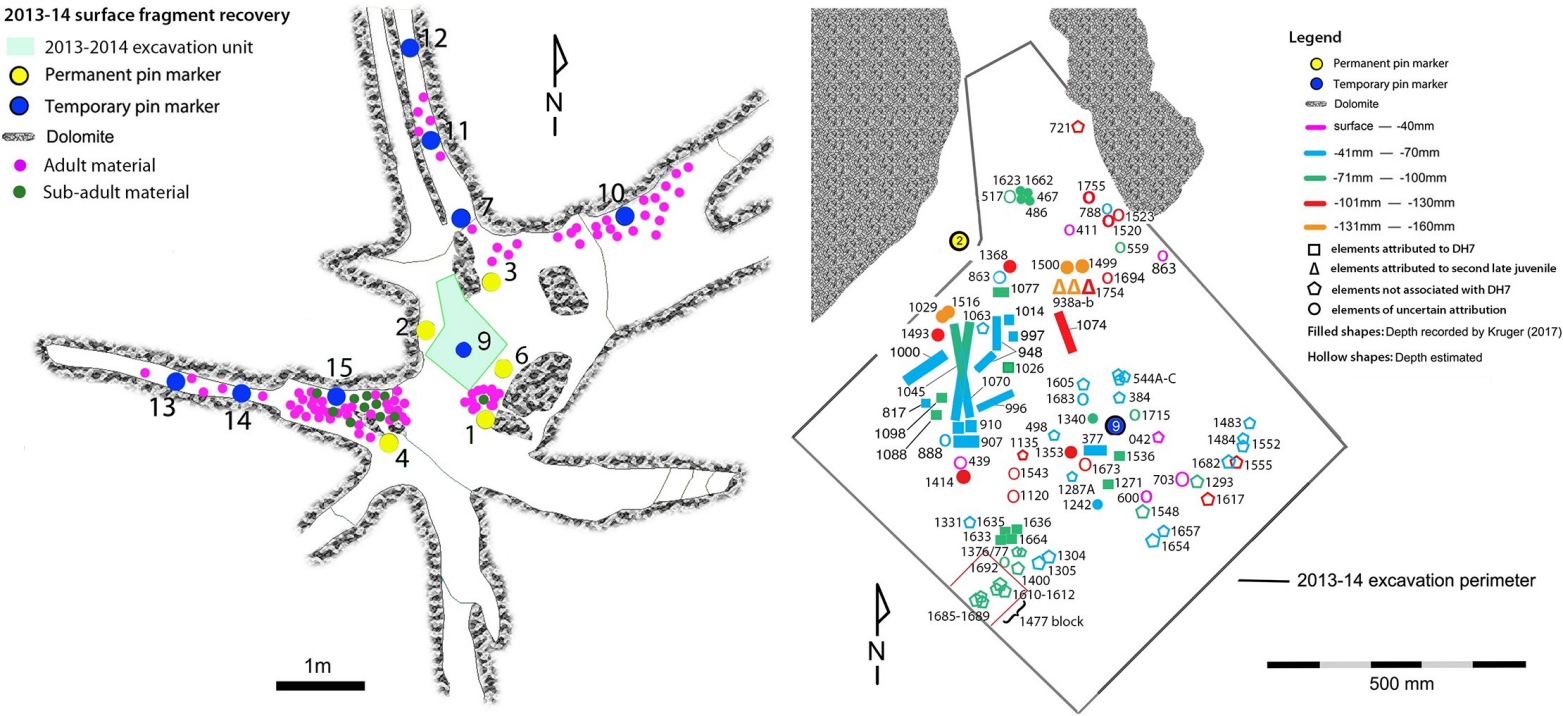
Fossil distribution. A) Schematic of the Dinaledi Chamber floor area, showing the distribution of adult and immature material recovered from the surface during the 2013–2014 expedition; B) All immature postcranial specimens recovered from the excavation pit by accession number and depth level.

When the Dinaledi Chamber was first assessed, approximately 300 fossil fragments were found scattered across the surface of the chamber floor (Fig 1A). The majority of the remaining assemblage came from a single excavation unit, 0.8 m^2^, excavated in two sessions in 2013 and 2014, to a depth of c20 cm below the surface. This unit lies midway inside the chamber and was targeted for excavation after fossil material was observed eroding through the surface. In total more than 1800 fossil specimens have been recovered from the Dinaledi Chamber.

Within the primary excavation unit, material is commingled. Detailed analyses of the taphonomy and distribution of the hominin bone accumulations suggested at least some of the bodies were deposited while still fleshed [11,17,18,19]. Further, the deposition of the remains appears to have occurred over a period of time, within different depth locations of the deposits [11]. The assemblage includes cranial and postcranial remains from both immature and adult individuals. Immature bone is less dense and less calcified than adult bone, and typically is less represented in assemblages than adult material [20,21]. Although two immature hemi-mandibles were recovered, in combination with a total of 32 deciduous teeth, immature cranial elements are extremely rare in the assemblage [10,22]. A mass of small fragments and sediment, removed *en bloc* in 2014 (U.W. 101–1477) appears to contain multiple fragments of an infant cranium. However, analysis of this thin and fragile material has only recently begun and identifications and descriptions are still underway.

While the comingled nature of the assemblage makes it challenging to associate skeletal parts to single individuals, several instances of postcranial elements in direct articulation have been noted [11,17,18]. This information, along with high-resolution data collected from the deposit [18,19], and anatomical assessment of the entire collection have made it possible for us to identify several portions of the skeleton of a single immature individual, which we designate as DH7.

**Fig 2:**
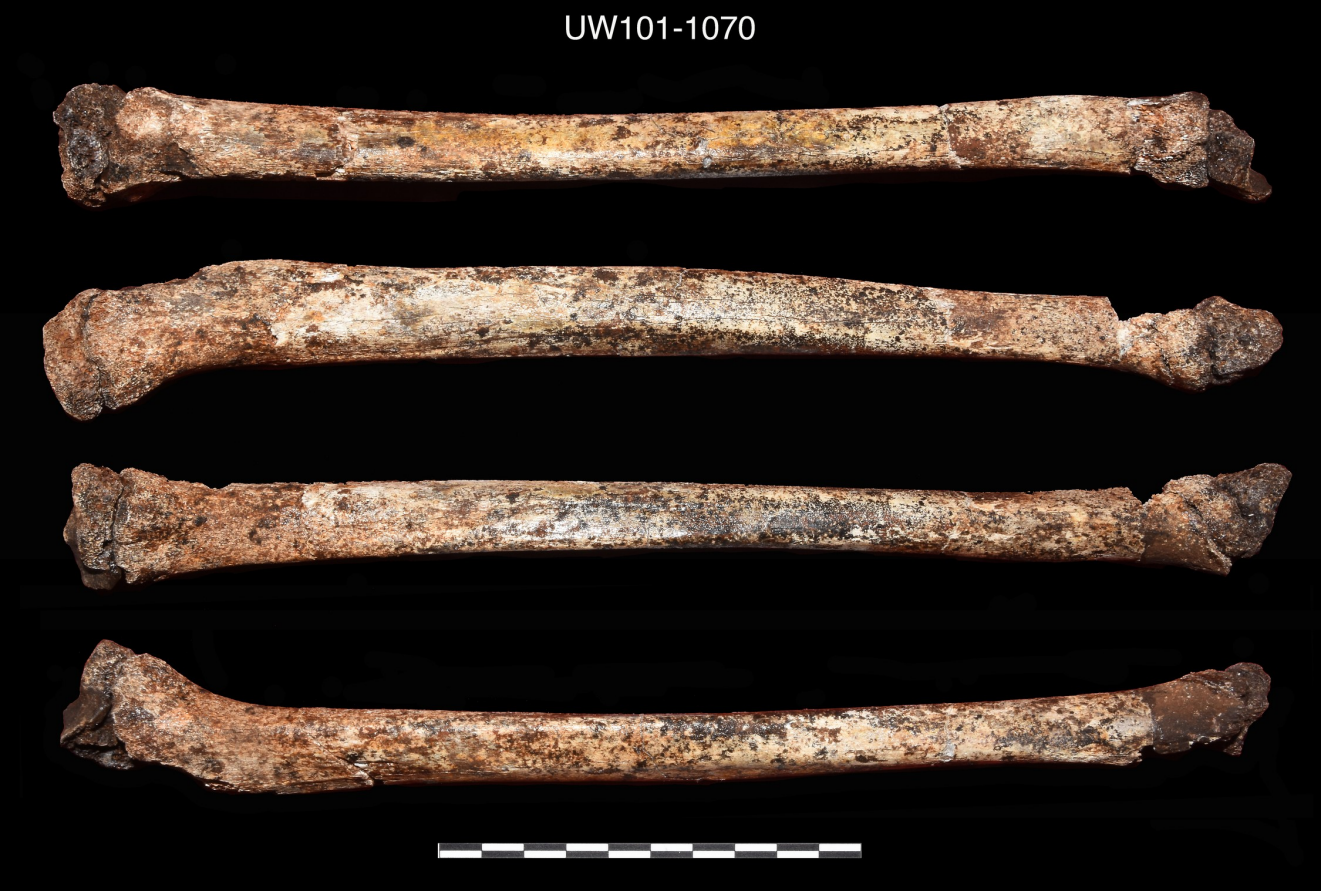
Left tibia, nearly complete (U.W. 101–1070). Note proximal and distal epiphyses affixed with paraloid.

**Fig 3:**
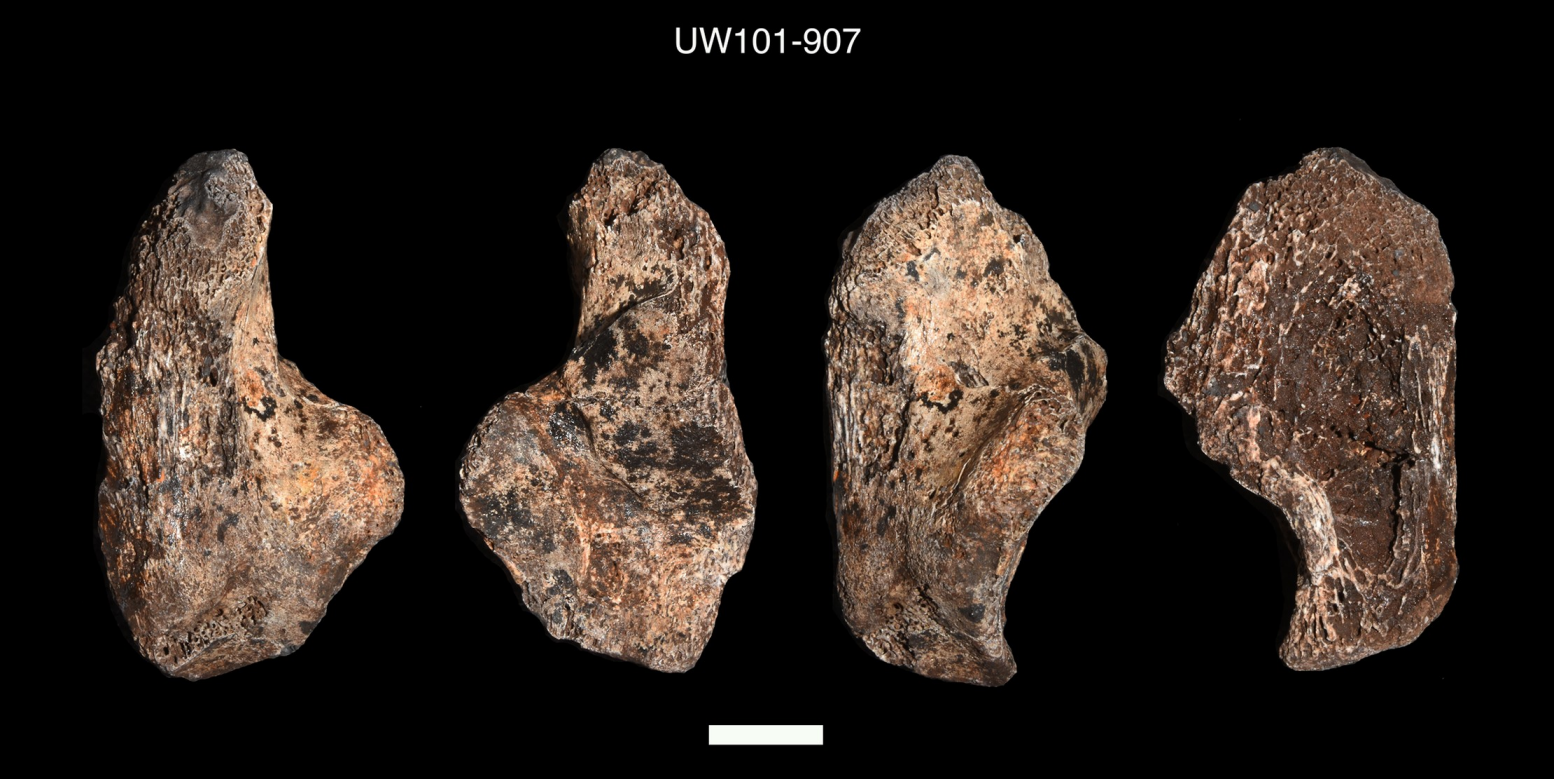
Left calcaneus (U.W. 101–907).

**Fig 4:**
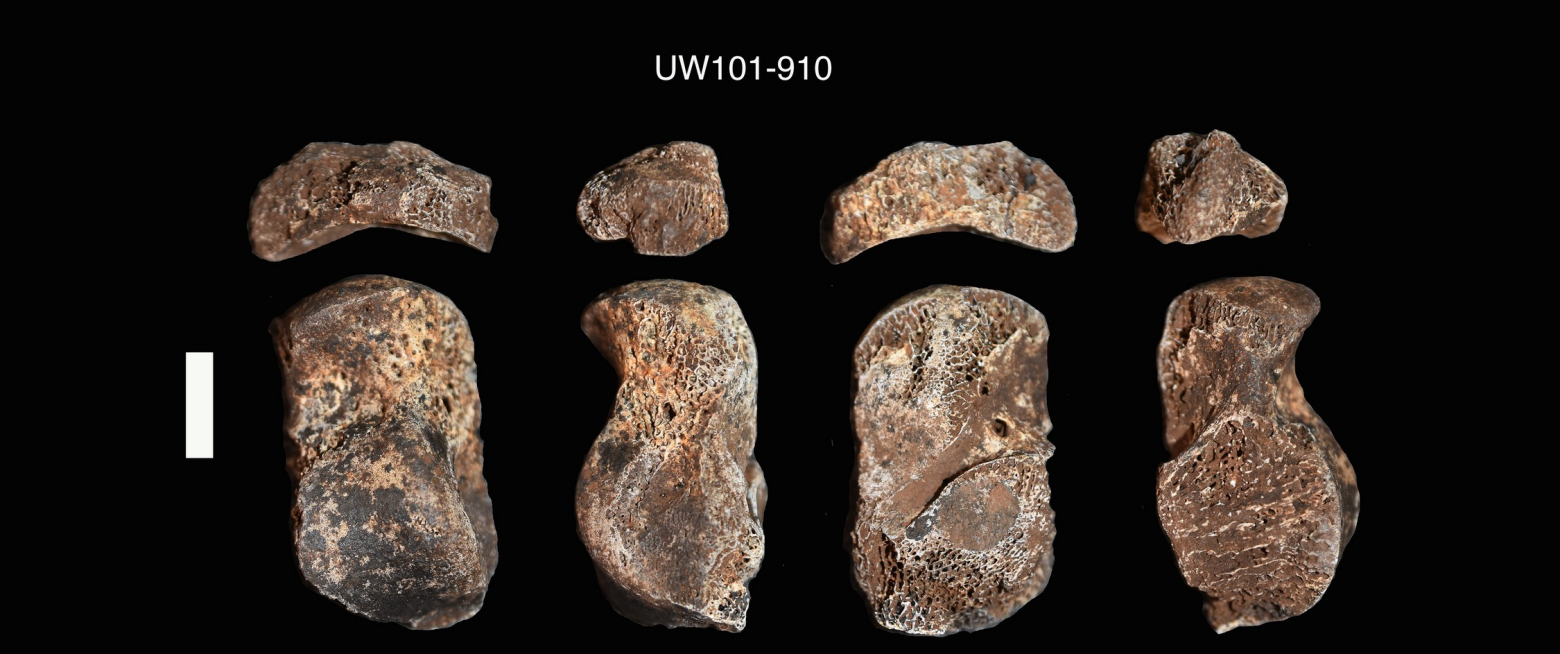
Left talus and navicular (U.W. 101–910).

**Fig 5:**
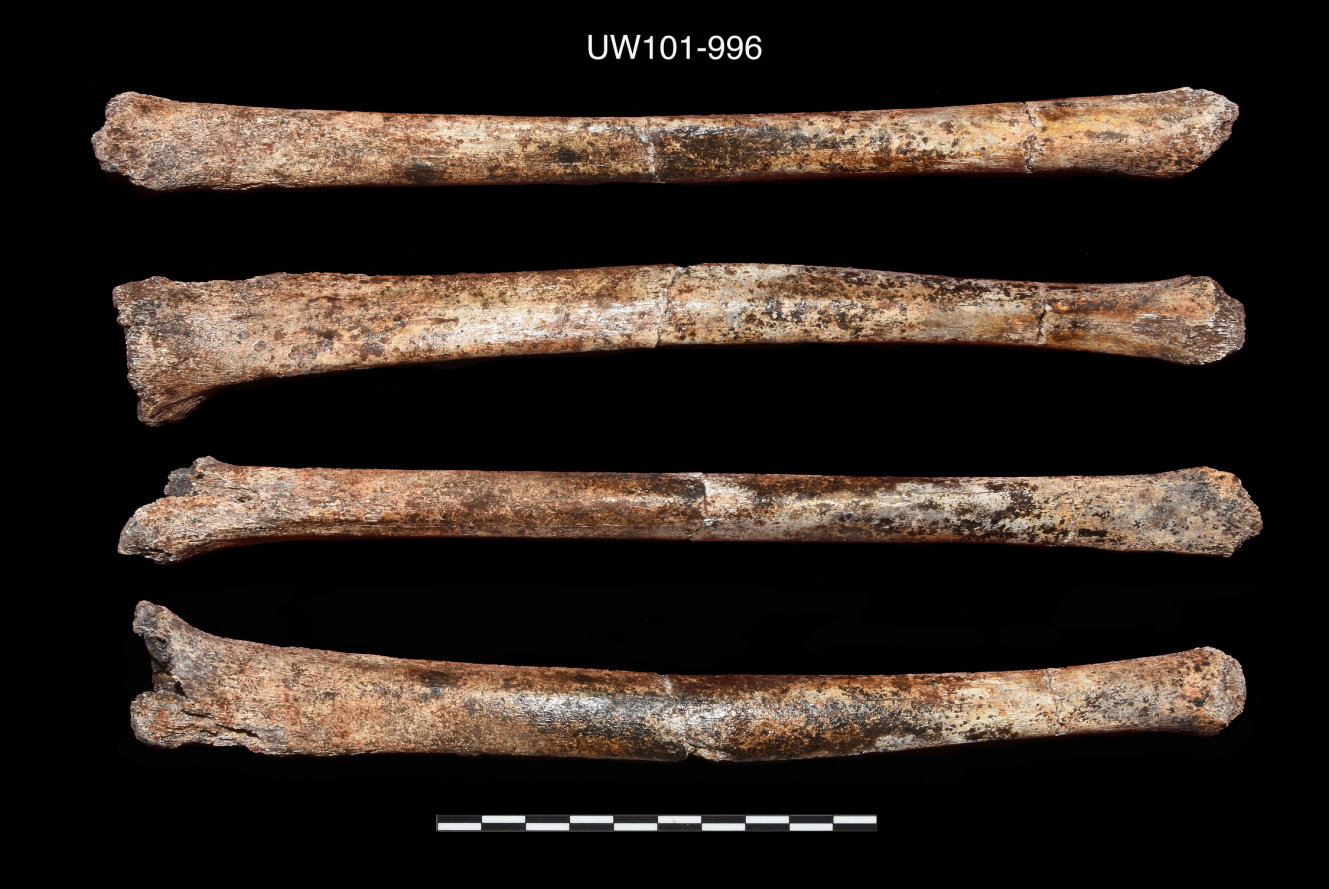
Right tibia, composite of three elements, missing epiphyses (U.W. 101–996).

Seventy-six postcranial specimens identified as ‘immature’ were recovered from the 2013–2014 Dinaledi excavations (Fig 1, Table 1). The excavation levels are divided into five levels: surface (surface to 40 mm), depth 1 (41–70 mm), depth 2 (71–100 mm), depth 3 (101–130 mm) and depth 4 (131–160 mm). DH7 is based upon *in situ* articulated elements excavated from the southwest portion of the excavation unit, approximately 41–100 mm below surface (depths 1 and 2). The articulated elements include an almost complete left tibia (U.W. 101–1070), recovered with unfused epiphyses in place (Fig 2); a partial left fibula (U.W. 101–1045), in three pieces, with heavily eroded surface morphology [23]; a left calcaneus (U.W. 101–907) (Fig 3); and a talus and navicular (recovered together as U.W. 101–910) (Fig 4). The three tarsals were originally designated “Foot 2” [24]. The preservation of the complete left tibia is noteworthy as we are familiar with no other specimen in the hominin fossil record that preserves epiphyses in place with this degree of surface detail. An immature right tibia, U.W. 101–996 (Fig 5) appears to be the antimere of the DH7 left tibia, U.W. 101–1070 [23]. This specimen is a composite of three fragments (U.W.101-996, U.W.101-1074 and U.W.101-1077), which were refit in the lab post-excavation, and accessioned to a single specimen number: U.W.101-996. All three fragments were recovered from depth 2 or 3, within 15 cm of the articulated left lower limb DH7 elements.

**Table 1:** Immature postcranial elements (n = 76) by catalogue number recovered from the 2013–2014 Dinaledi Chamber excavations. Descriptions in [12,17,23,24,34,35,36].

	Catalogue no.	Description
1.	U.W. 101–014	Unsided femur shaft, fragment
2.	U.W. 101–031	Right humerus, distal and partial shaft
3.	U.W. 101–042	Unsided tibia, shaft fragment
4.	U.W. 101–080	Left talus
5.	U.W. 101–244	Left metatarsal 1
6.	U.W. 101–248	Left metatarsal 4, partial
7.	U.W. 101–259	Right radius, proximal and partial shaft
8.	U.W. 101–411	Left humerus, shaft fragment
9.	U.W. 101–439	Thoracic vertebra, fragment of neural arch
10.	U.W. 101–467	Right cuboid (also published as U.W. 101–487)
11.	U.W. 101–486	Right ilium, fragment
12.	U.W. 101–498	Left tibia, proximal shaft
13.	U.W. 101–517	Right metacarpal 3
14.	U.W. 101–559	Left metacarpal 3
15.	U.W. 101–600	Left ulna, proximal fragment (possibly immature)
16.	U.W.101-634	Unsided bone shaft, fragment
17.	U.W. 101–703	Left radius, proximal
18.	U.W. 101–721	Right metacarpal 3, distal epiphysis
19.	U.W. 101–788	Long bone epiphysis, partial
20.	U.W. 101–817	Left fibula, proximal
21.	U.W. 101–863	Unsided long bone (either femur or humerus)
22.	U.W. 101–888	Coccyx (possibly immature)
23.	U.W. 101–907	Left calcaneus
24.	U.W. 101–910	Left talus + navicular (two elements under one catalogue number)
25.	U.W. 101-938a	Right femur, partial shaft
26.	U.W. 101-938b	Right femur, partial head epiphysis
27.	U.W. 101–948	Right humerus
28.	U.W. 101–964	Right ulna, proximal
29.	U.W. 101–996	Right tibia
30.	U.W. 101–997	Right navicular
31.	U.W. 101–1000	Right femur, proximal
32.	U.W. 101–1026	Metacarpal, head epiphysis
33.	U.W. 101–1029	Unsided metacarpal shaft
34.	U.W. 101–1045	Left fibula, in three fragments (a, b, and c) that do not refit
35.	U.W. 101–1070	Left tibia, with proximal and distal epiphyses
36.	U.W. 101–1088	Right ischium, partial
37.	U.W. 101–1098	Right femur epiphysis
38.	U.W. 101–1120	Left femur, distal
39.	U.W. 101–1229	Right clavicle, partial shaft
40.	U.W. 101–1242	Right cuneiform, intermediate
41.	U.W. 101–1271	Unsided metacarpal, shaft fragment
42.	U.W. 101–1293	Unsided ulna, midshaft fragment
43.	U.W. 101–1340	Unsided humerus, shaft
44.	U.W. 101–1353	Right scapula, lateral spine fragment (possibly immature)
45.	U.W. 101–1368	Right metatarsal 4
46.	U.W. 101–1414	Left ischium, partial
47.	U.W. 101–1483	Right hallucal phalanx epiphysis, proximal
48.	U.W. 101–1484	Right pedal phalanx, middle
49.	U.W. 101–1493	Thoracic (upper) vertebra neural arch, fragment
50.	U.W. 101–1499	Right metatarsal 2 and metatarsal 1 epiphysis (two elements under one catalogue number)
51.	U.W. 101–1500	Right metatarsal 3
52.	U.W. 101–1516	Unsided manual phalanx, proximal shaft
53.	U.W. 101–1520	Fibula, shaft fragment (possibly immature)
54.	U.W. 101–1523	Right femur shaft, distal
55.	U.W. 101–1536	Unsided metacarpal, shaft fragment
56.	U.W. 101–1543	Unsided humeral head epiphysis, partial
57.	U.W.101-1552	Unsided proximal epiphysis of proximal manual phalanx
58.	U.W. 101–1555	Left femur, proximal and unfused epiphysis
59.	U.W. 101–1617	Unsided calcaneus epiphysis
60.	U.W. 101–1623	Right talus
61.	U.W. 101–1633	Unsided metacarpal, shaft fragment
62.	U.W. 101–1635	Unsided manual phalanx, proximal shaft
63.	U.W. 101–1636	Unsided metacarpal, shaft fragment
64.	U.W. 101–1654	Unsided metacarpal, shaft distal fragment
65.	U.W. 101–1657	Unsided pedal phalanx, proximal
66.	U.W. 101–1662	Right calcaneus, fragmentary
67.	U.W. 101–1664	Unsided manual phalanx, intermediate distal fragment
68.	U.W. 101–1673	Cervical vertebra, neural arch fragment
69.	U.W. 101–1682	Left intermediate cuneiform (possibly immature)
70.	U.W. 101–1683	Unsided femur epiphysis, distal fragment
71.	U.W. 101–1692	Cervical (axis) vertebra, odontoid process
72.	U.W. 101–1694	Right femur epiphysis, distal lateral condyle
73.	U.W. 101–1715	Unsided metacarpal head epiphysis
74.	U.W.101-1754/1755	Left ischium, in two parts

Considering the number of immature skeletal material recovered from the Dinaledi hominin deposit (Table 1), we set out to test the hypothesis that additional elements of this partial skeleton were present in the sample.

## Materials and methods

We assess 70 immature skeletal remains from the 2013–2104 season to identify materials that may belong to the proposed DH7 individual comprised of the six lower limb elements. All fossil analyzed in this study are from the Dinaledi Chamber, accession numbers U.W. 101- (See Table 1). *H*. *naledi* original fossils are curated at the Evolutionary Studies Institute, University of the Witwatersrand in Johannesburg, South Africa, and researchers may apply to study them through the Fossil Access Committee by contacting the University Curator for Fossil and Rock Collections. Additionally, 3D surface models and images of DH7 elements are available for public access and download at Morphosource.org (See Table 3). The South African Heritage Resource Agency and Cradle of Humankind UNESCO World Heritage Site Management Authority granted the permits to work on the Rising Star site, excavation permit ID: 952. All necessary permissions were obtained for the described study, which complied with all relevant regulations.

**Fig 6:**
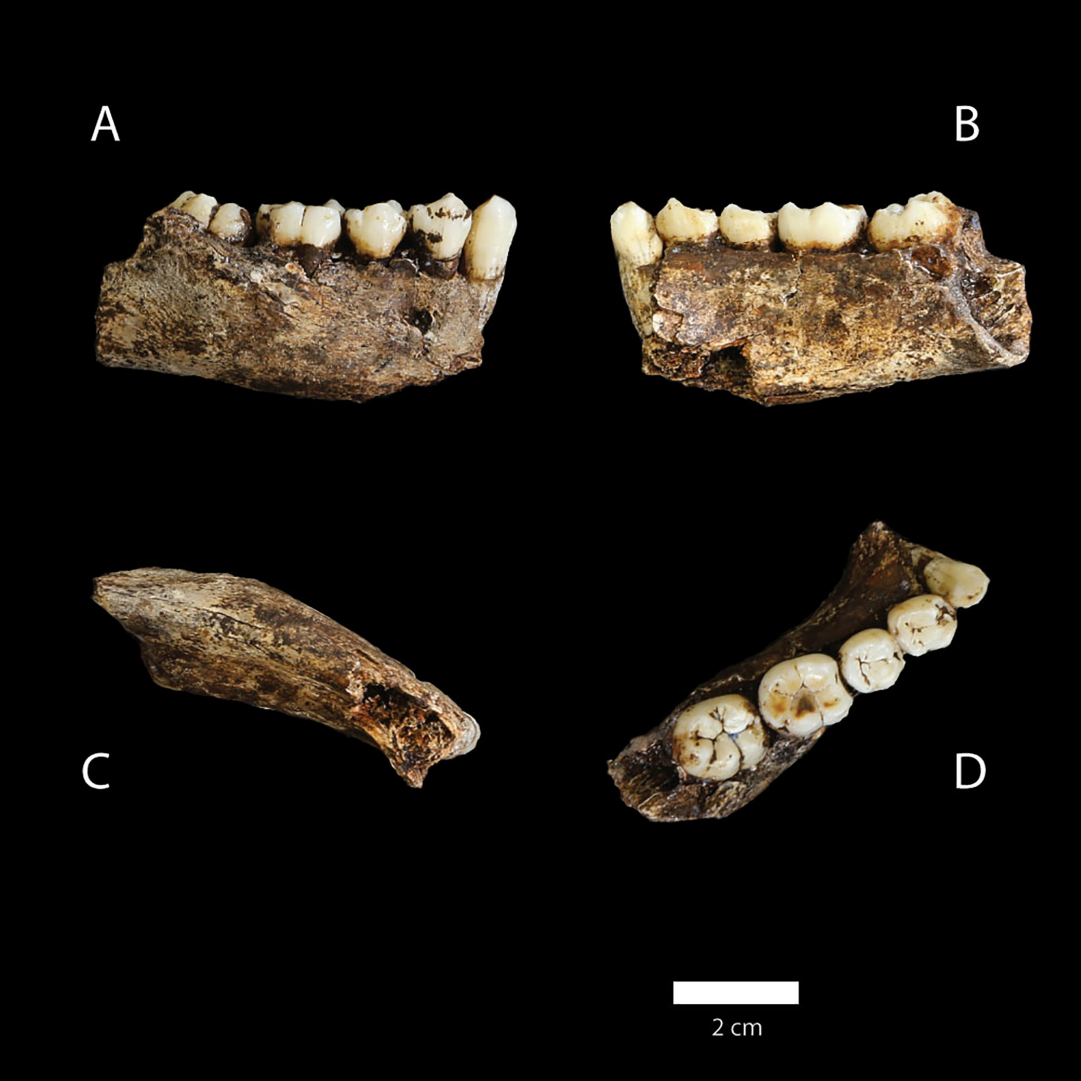
U.W. 101–377 right hemi-mandible. Right hemi-mandible with permanent teeth C, P3, P4, M1 and M2; canine U.W. 101–1014 refit in lab.

A minimum number of individuals (MNI) was previously assessed from the 2013–2104 season based on dental remains: nine immature individuals, and six adult individuals [10,13] (Table 2). Six of the dental individuals are either ‘Infants’ (deciduous dentition only) or ‘Early Juveniles’ (with first molars erupted). Only a single dental specimen represents a ‘Late Juvenile’ with second molars erupted [13] (Table 2). This ‘Late Juvenile’ is represented by a right mandibular fragment, U.W. 101–377, preserving permanent teeth C, P3, P4, M1 and M2 (canine U.W. 101–1014 refit in lab) [10,11,13,20] (Fig 6). The partial mandible was recovered from depth 1 in the excavation unit.

**Table 2:** *Homo naledi* age classes for 13 individuals represented in 2013–2014 excavation season, based on dentition. Two additional individuals are present based on MNI, but only approximate age can be determined: One is an adult, and one is an immature [13].

Dentition Present	Age Class	Number in assemblage
Deciduous dentition only	Infant	3
First molar(s)	Early juvenile	3
Second molar(s)	Late juvenile	1
Third molar(s)—unworn / partially erupted	Sub-Adult	1
All permanent teeth fully erupted, little to moderate wear	Young Adult	4
All permanent teeth fully erupted, heavy wear	Old Adult	1

The DH7 associated lower limb represents a ‘Late Juvenile’ in skeletal maturation stage, with unfused but well-developed epiphyses of the hip, knee and ankle, a maturational skeletal pattern that is consistent across apes and hominins [13,25–29].

Criteria for including or excluding elements from association with DH7 include: duplication of element; developmental stage; size difference; spatial proximity (depth and span); refitting of specimens into a single element; and articulation/anatomical position of elements (Table 3). Skeletal maturation continuity was assessed using established standards from the literature [30–33]; cf [5]. Evaluations were made using the original fossils, original excavation notes and photographs, and published descriptions of *H*. *naledi* anatomy [17,23,24,34,35,36]. The vertical and horizontal context of immature material within the excavation unit was also used to help identify potentially associated elements [19]. If depth location was not available from the previously collected 3D data [18,19], depth was estimated based on excavation photographs and comparisons with known-depth specimens.

**Table 3:** List of specimens recovered from the 2013–2014 Dinaledi Chamber excavations that have been identified as immature. Specimens attributed to DH7 are highlighted in grey. Criteria for determining elements associated with DH7: 1 Duplication of element, 2 Different developmental stage, 3 Size difference, 4 Spatial proximity, 5 Refitting of specimens into single element, 6 Articulation/anatomical position of elements.

	Catalogue no.	Description	Reject or accept association w DH7 on criteria
1.	U.W. 101–014	Unsided femur shaft, fragment	REJECT on #4
2.	U.W. 101–031	Right humerus, distal and partial shaft	REJECT on #1 and #4
3.	U.W. 101–042	Unsided tibia, shaft fragment	REJECT on #1
4.	U.W. 101–080	Left talus	REJECT on #1 and #4
5.	U.W. 101–244	Left metatarsal 1	REJECT on #2 and #4 [24]
6.	U.W. 101–248	Left metatarsal 4, partial	REJECT on #2 and #4 [24]
7.	U.W. 101–259	Right radius, proximal and partial shaft	REJECT on #4
8.	U.W. 101–377; U.W. 101–1014	Right hemi-mandible with C, P3, P4, M1 and M2 with canine U.W. 101–1014 refit in lab	ACCEPT on #2, #4 and #5
9.	U.W. 101–411	Left humerus, shaft fragment	REJECT on #4
10.	U.W. 101–439	Thoracic vertebra, fragment of neural arch	Indeterminate; fragment whose developmental stage uncertain; Possible REJECT on #4
11.	U.W. 101–467	Right cuboid (also published as U.W. 101–487)	REJECT on #2, #3 and #4 [24]
12.	U.W. 101–486	Right ilium, fragment	REJECT on #3 and #4
13.	U.W. 101–498	Left tibia, proximal shaft	REJECT on #1
14.	U.W. 101–517	Right metacarpal 3	REJECT on #2, #3 and #4 [17]
15.	U.W. 101–559	Left metacarpal 3	REJECT on #2, #3 and #4 [17]
16.	U.W. 101–600	Left ulna, proximal fragment (possibly immature)	Indeterminate; fragment whose developmental stage uncertain; Possible REJECT on #4
17.	U.W.101-634	Unsided bone shaft, fragment	REJECT on #4
18.	U.W. 101–703	Left radius, proximal	REJECT on #2 and #4
19.	U.W. 101–721	Right metacarpal 3, distal epiphysis	REJECT on #2, #3 and #4 [17]
20.	U.W. 101–788	Long bone epiphysis, partial	REJECT on #4
21.	U.W. 101–817	Left fibula, distal	ACCEPT on #4 and #5
22.	U.W. 101–863	Unsided long bone (either femur or humerus)	REJECT on #2 and #4
23.	U.W. 101–888	Coccyx (possibly immature)	Indeterminate; fragment whose developmental stage uncertain
24.	U.W. 101–907	Left calcaneus	ACCEPT on #4 and #6
25.	U.W. 101–910	Left talus + navicular (two elements under one catalogue number)	ACCEPT on #4 and #6
26.	U.W. 101-938a	Right femur, partial shaft	REJECT on #1 and #4
27.	U.W. 101-938b	Right femur, partial head epiphysis	REJECT on #1 and #4
28.	U.W. 101–948	Right humerus	ACCEPT on #2, #3, and #4
29.	U.W. 101–964	Right ulna, proximal	REJECT on #4
30.	U.W. 101–996	Right tibia	ACCEPT on #2, #3 and #4 [23]
31.	U.W. 101–997	Right navicular	REJECT on #2 and #3 [24]
32.	U.W. 101–1000	Right femur, proximal	ACCEPT on #2, #3 and #4
33.	U.W. 101–1026	Metacarpal, head epiphysis	REJECT on #2
34.	U.W. 101–1029	Unsided metacarpal shaft	Indeterminate; Possible ACCEPT on #2 and #3 [17]; Possible REJECT on #4
35.	U.W. 101–1045	Left fibula, in three fragments (a, b, and c) that do not refit	ACCEPT on #2, #3, #4 and #6
36.	U.W. 101–1070	Left tibia, with proximal and distal epiphyses	ACCEPT on #2, #3, #4 and #6 [23]
37.	U.W. 101–1088	Right ischium, partial	ACCEPT on #2, #3, and #4
38.	U.W. 101–1098	Right femur epiphysis	ACCEPT on #2, #4 and #6
39.	U.W. 101–1120	Left femur, distal	REJECT on #2
40.	U.W. 101–1229	Right clavicle, partial shaft	REJECT on #2 and #3 [34]
41.	U.W. 101–1242	Right cuneiform, intermediate	REJECT on #2, #3 and #4 [24]
42.	U.W. 101–1271	Unsided metacarpal, shaft fragment	ACCEPT on #2 and #3 [17]
43.	U.W. 101–1293	Unsided ulna, midshaft fragment	Indeterminate; fragment whose developmental stage uncertain
44.	U.W. 101–1340	Unsided humerus, shaft	Indeterminate; fragment whose developmental stage uncertain
45.	U.W. 101–1353	Right scapula, lateral spine fragment (possibly immature)	Indeterminate; fragment whose developmental stage uncertain
46.	U.W. 101–1368	Right metatarsal 4	REJECT on #2, #3 and #4 [24]
47.	U.W. 101–1400	Left mandibular fragment with Ldc1-LM1 and L12 germ	REJECT on #2 [13]
48.	U.W. 101–1414	Left ischium, partial	REJECT on #2 [36]
49.	U.W.101-1477	Multiple fragile fragments, including cranial and vertebral bits, removed en bloc	REJECT on #2 and #3
50.	U.W. 101–1483	Right hallucal phalanx epiphysis, proximal	REJECT on #2, #3 and #4 [24]
51.	U.W. 101–1484	Right pedal phalanx, middle	REJECT on #2, #3 and #4 [24]
52.	U.W. 101–1493	Thoracic (upper) vertebra neural arch, fragment	Indeterminate; fragment whose developmental stage uncertain; Possible ACCEPT on #4
53.	U.W. 101–1499	Right metatarsal 2 and metatarsal 1 epiphysis (two elements under one catalogue number)	REJECT on #2, #3 and #4 [24]
54.	U.W. 101–1500	Right metatarsal 3	REJECT on #2, #3 and #4 [24]
55.	U.W. 101–1516	Unsided manual phalanx, proximal shaft	Indeterminate; fragment heavily eroded whose developmental stage uncertain; Possible ACCEPT on #4
56.	U.W. 101–1520	Fibula, shaft fragment (possibly immature)	Indeterminate; fragment whose developmental stage uncertain; Possible REJECT on #4 [23]
57.	U.W. 101–1523	Right femur shaft, distal	REJECT on #3 and #4 [23]
58.	U.W. 101–1536	Unsided metacarpal, shaft fragment	ACCEPT on #2, #3 and #4 [17]
59.	U.W. 101–1543	Unsided humeral head epiphysis, partial	REJECT on #2
60.	U.W.101-1552	Unsided proximal epiphysis of proximal manual phalanx	REJECT on #3 and #4 [17]
61.	U.W. 101–1555	Left femur, proximal and unfused epiphysis	Indeterminate; heavily eroded [23]; Possible REJECT on #4
62.	U.W. 101–1617	Unsided calcaneus epiphysis	Indeterminate; fragment whose provenience uncertain; Possible REJECT on #3
63.	U.W. 101–1623	Right talus	REJECT on #2, #3 and #4 [24]
64.	U.W. 101–1633	Unsided metacarpal, shaft fragment	ACCEPT on #2, #3 and #4 [17]
65.	U.W. 101–1635	Unsided manual phalanx, proximal shaft	ACCEPT on #2, #3 and #4 [17]
66.	U.W. 101–1636	Unsided metacarpal, shaft fragment	ACCEPT on #2, #3 and #4 [17]
67.	U.W. 101–1654	Unsided metacarpal, shaft distal fragment	Indeterminate; Possible ACCEPT on #2 and #3 [17]; Possible REJECT on #4
68.	U.W. 101–1657	Unsided pedal phalanx, proximal	Indeterminate; Possible REJECT on #2, #3 and #4 [24]
69.	U.W. 101–1662	Right calcaneus, fragmentary	REJECT on #2, #3 and #4 [24]
70.	U.W. 101–1664	Unsided manual phalanx, intermediate distal fragment	ACCEPT on #2, #3 and #4 [17]
71.	U.W. 101–1673	Cervical vertebra, neural arch fragment	Indeterminate; fragment whose developmental stage uncertain
72.	U.W. 101–1682	Left intermediate cuneiform (possibly immature)	Indeterminate; developmental stage uncertain
73.	U.W. 101–1683	Unsided femur epiphysis, distal fragment	Indeterminate; fragment
74.	U.W. 101–1692	Cervical (axis) vertebra, odontoid process	REJECT on #2 and #3 [35]
75.	U.W. 101–1694	Right femur epiphysis, distal lateral condyle	Indeterminate; fragment eroded; Possible REJECT on #2 and #4
76.	U.W. 101–1715	Unsided metacarpal head epiphysis	REJECT on #2
77.	U.W.101-1754/1755	Left ischium, in two parts	REJECT #4, #5 and #6

In this commingled sample we set a very high evidentiary standard to accept the association of different elements. Anatomical *in situ* articulation of the left lower limb elements provides certain evidence that these elements represent a single individual, deposited with soft tissue still intact. The U.W. 101–996 tibia meets this standard on the evidence of its mirror-image anatomy to U.W. 101–1070 [23], its identical state of epiphyseal development, and its very close spatial and depth position to the articulated left lower limb elements of DH7. The discovery that all fragments of two antimere elements are very near each other may suggest a low degree of disaggregation of skeletal elements in this part of the deposit.

The evidence for skeletal association here meets the standards linking immature partial skeletons at other hominin sites with commingled individuals [1,4,8,9]. El Sidrón J1 is an Early Juvenile partial skeleton (*H*. *neanderthalensis*) recovered mostly from a 1 m^2^ unit, together with multiple elements from at least one younger and one older immature individual. The consistent developmental stage and some direct articulations support the interpretation that the J1 material represents a single individual [1]. The MH1 immature partial skeleton from Malapa also includes some elements in direct anatomical articulation [4], and of consistent developmental stage, although one adult partial skeleton and another immature individual are known to be present in this assemblage. A sub-adult partial skeleton from Dmanisi, Georgia is evidenced based upon stratigraphic association, with all elements found within a 1 m stratum across 7 m^2^, along with developmental consistency and lack of element duplication [8,9]. As in the Dinaledi example, the recovery of more skeletal material from any of these active sites may necessitate revisions to the original hypothesis of association.

The 2013–2014 Dinaledi excavations involved a single 0.8 m^2^ unit, in addition to a small amount of material collected from the chamber surface. While the surface area of the chamber consists of approximately 20 m^2^, the depth of the sediments within is currently unknown. Consequently, we cannot predict what other remains of this individual may yet be recovered. We further caution that many elements in the collection are too fragmentary to accurately assess developmental stage.

## Results

**Fig 7:**
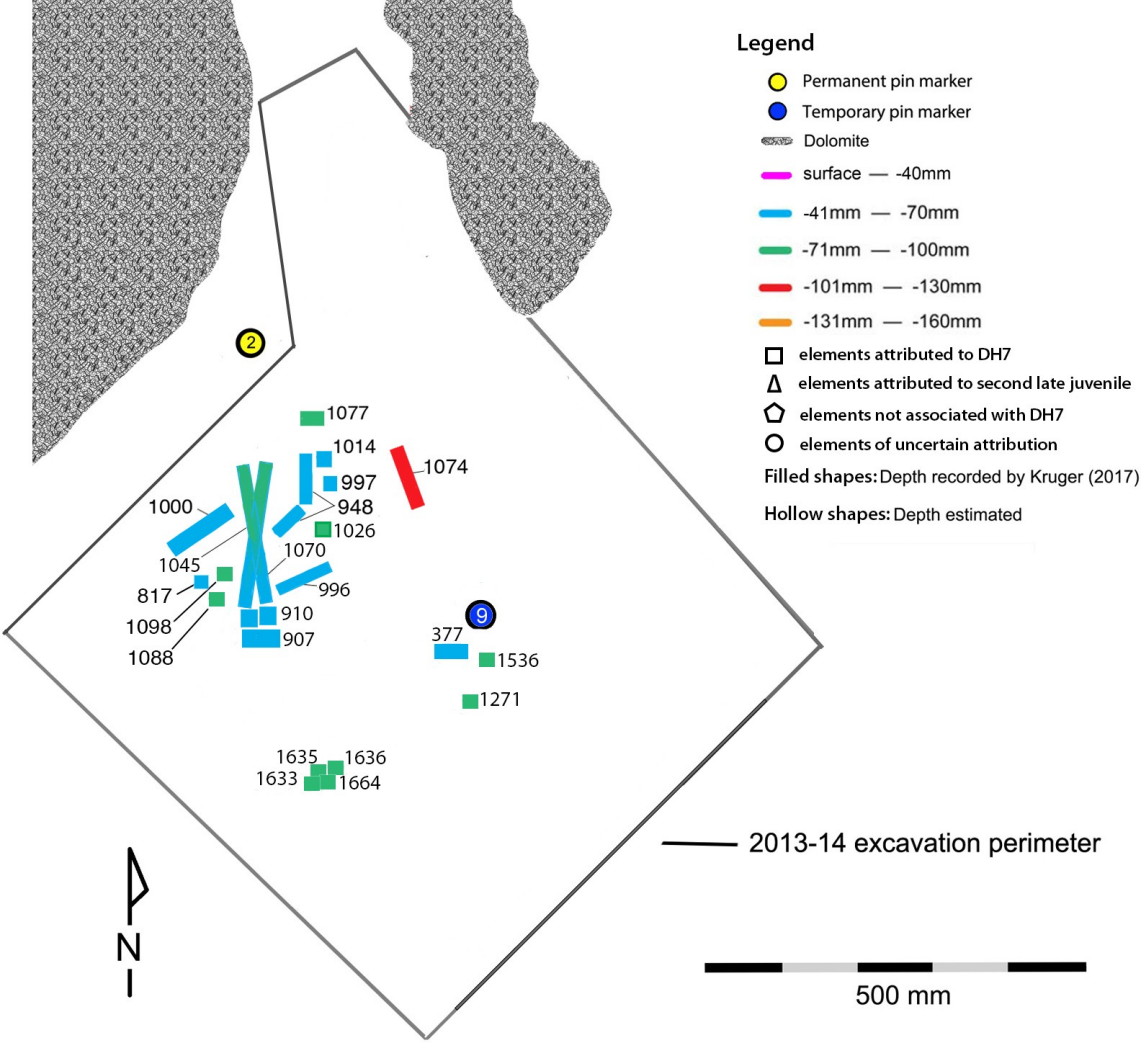
Distribution and location map of the elements associated with DH7.

**Fig 8:**
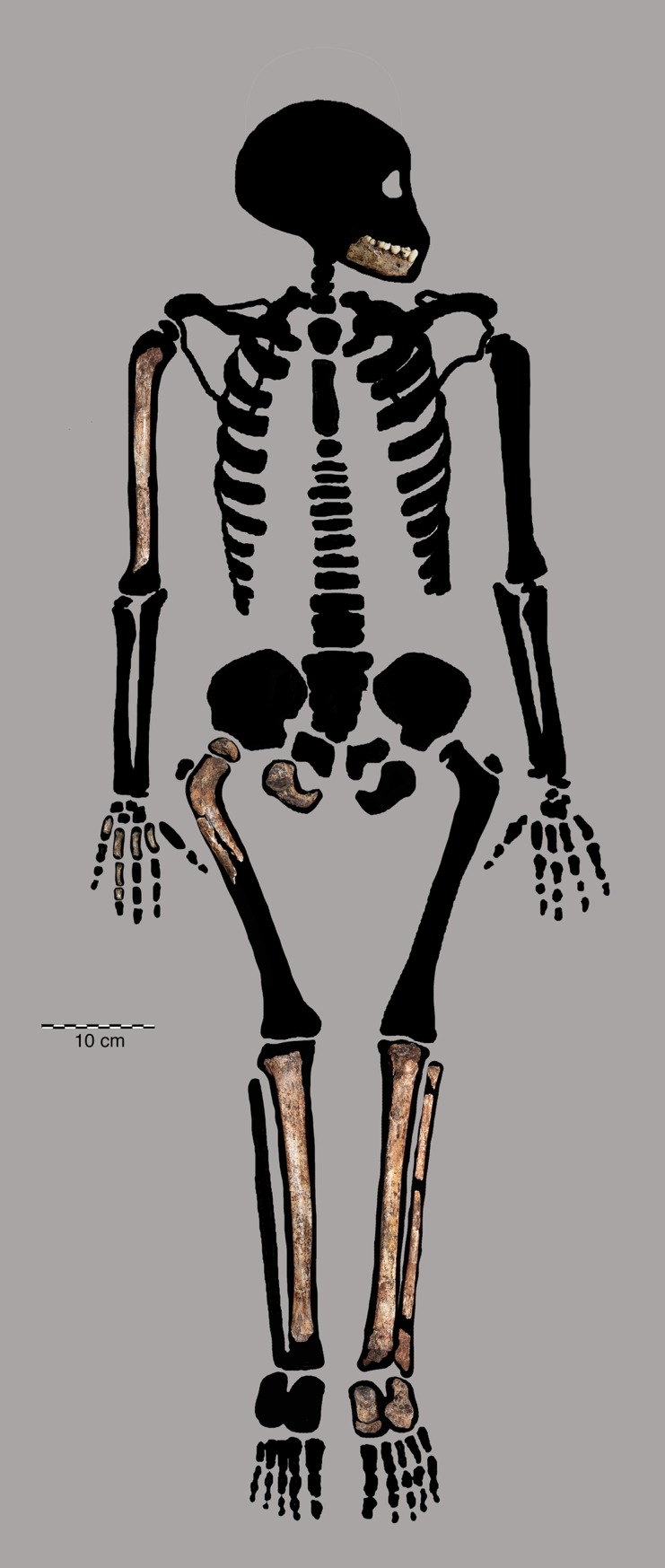
DH7 partial skeleton.

We identified additional skeletal specimens that are developmentally consistent in maturity indicators with the remains of DH7, recovered within 2–20 cm of the articulated lower limb (Figs 7 and 8; Table 4). Consistent with the dental evidence from the Dinaledi Chamber of the ‘Late Juvenile’ age class, postcranial elements that demonstrate a ‘Late Juvenile’ stage of maturation are quite rare within the sample.

**Table 4:** Elements associated with immature juvenile skeleton DH7.

Catalogue no.	Description
U.W. 101–377; U.W. 101–1014	Right hemi-mandible with C, P3, P4, M1 and M2 with canine U.W. 101–1014 refit in lab [10,13,22].
U.W. 101–817	Left fibula, distal element, broken and excavated nearby but separately from fibula shaft U.W.101-1045. The specimen has metaphyseal surface along the articular areas [23].
U.W. 101–907	Left calcaneus. The specimen retains a small region of metaphyseal surface on the posterior, medial aspect. It is small in size compared to other adult calcaneal bones in the assemblage [24].
U.W. 101–910	Left talus + navicular (two elements under one catalogue number). Both elements are small in size compared to other adult tarsal bones in the Dinaledi assemblage, and “lack the defined borders” of adult tarsals [24, 27].
U.W. 101–948	Right humerus comprised of two shaft elements conjoined in the lab. Both the proximal and distal ends are less well preserved. Proximally the humeral head has exposed metaphyseal surface indicating no fusion of an epiphysis. There is possibly a minute metaphyseal surface on the most lateral distal olecranon region [34].
U.W. 101–996	Right tibia, missing the epiphyses and with the ends of the shaft somewhat damaged. Antimere U.W.101-1070 [23].
U.W. 101–1000	Right femur, proximal. The proximal femur is not well preserved. There are splintered fragments missing from the shaft, and the greater and lesser trochanters are not preserved [23].
U.W. 101–1045	Left fibula composed of three fragments that do not refit. The fragments are eroded, and moderate damage prevents characterization of surface features [23]. Immature based on size [23] and in situ association with immature tibia U.W.101-1070.
U.W. 101–1070	Left tibia, with proximal and distal epiphyses. The proximal epiphysis of this specimen appears almost fully formed in size and shape, and is well-fitted to the diaphysis, although unfused. The distal epiphysis appears less formed in adult morphology, but is also less well preserved [23].
U.W. 101–1088	Right ischium, partial. The posterior acetabular epiphysis is fully fused to form a continuous lunate surface. The acetabular surfaces and the ischial tuberosity epiphysis are unfused, evident by beveling and rough surfaces. The pubic ramus fusion information is not preserved [36].
U.W. 101–1098	Right femur epiphysis, proximal. The partial femoral head is unfused but mature in size and appearance, with a well-formed fovea, a smooth surface, and an angulated margin with a “beak-like” projection similar to pre-adolescent human head epiphyses [23].
U.W. 101–1271	Unsided metacarpal, shaft fragment. Metacarpal shaft fragment is pre-adult in size, but age-consistent with the other hand materials combined into “Hand 5” [15]. Shaft exhibit defined adult morphology of a distinctive palmar curvature.
U.W. 101–1536	Unsided metacarpal, shaft fragment only [17]. Shaft fragment is pre-adult in size, but exhibits defined features of metacarpal, with palmar curvature and a slight ridging on the palmar aspect.
U.W. 101–1633	Unsided metacarpal, shaft fragment. Metacarpal shaft fragment is pre-adult in size, but exhibit defined morphology indicative of older juvenile [17].
U.W. 101–1635	Unsided manual proximal phalanx, distal shaft. Distinctive in shape with distal bicondylar ridging, although bone weathered. Missing proximal end of bone. Shaft is pre-adult in size [17].
U.W. 101–1636	Unsided metacarpal, shaft fragment. Metacarpal shaft fragment is pre-adult in size, but exhibits distinctive palmar curvature [17].
U.W. 101–1664	Unsided manual phalanx, intermediate distal fragment. Manual phalangeal shaft fragment is pre-adult in size, but consistent developmentally with other associated hand bones [17].

**Fig 9:**
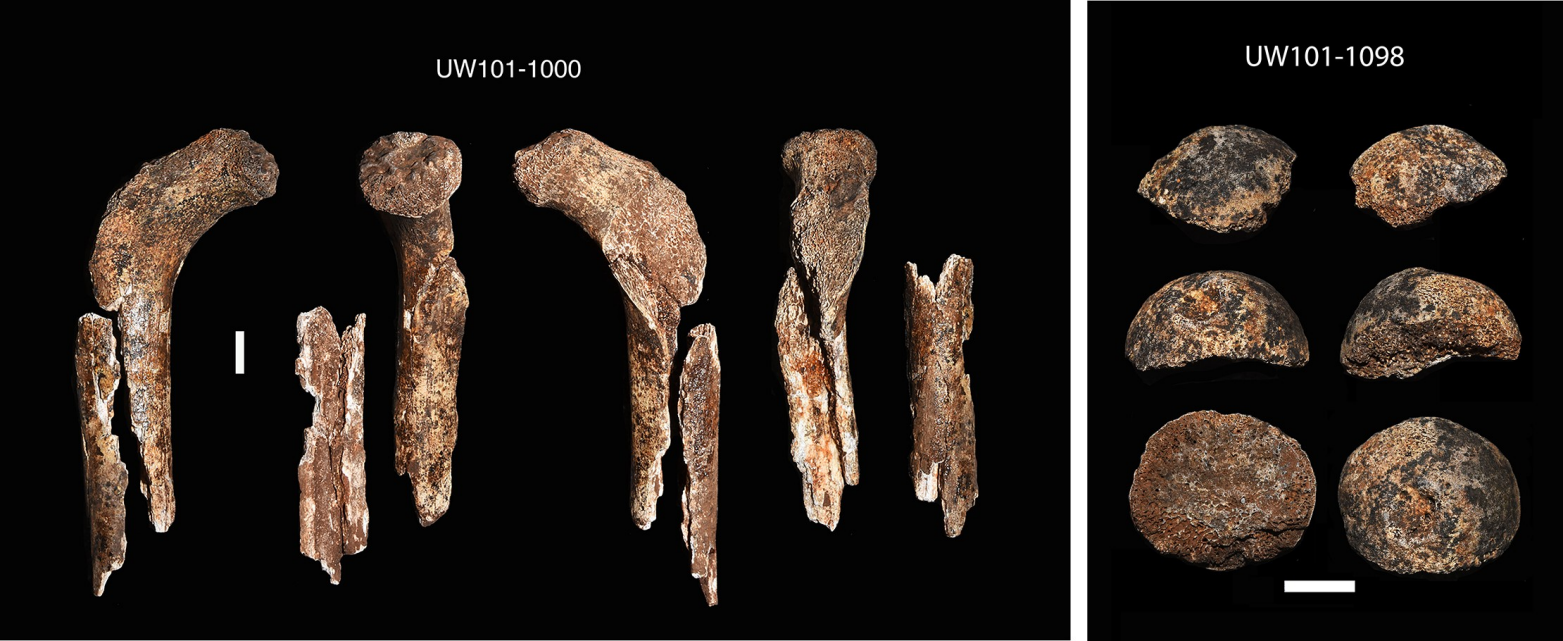
Right femur. a) Proximal fragment with metaphyseal surface (U.W. 101–1000); b) unfused femoral head epiphysis (U.W. 101–1098).

**Fig 10:**
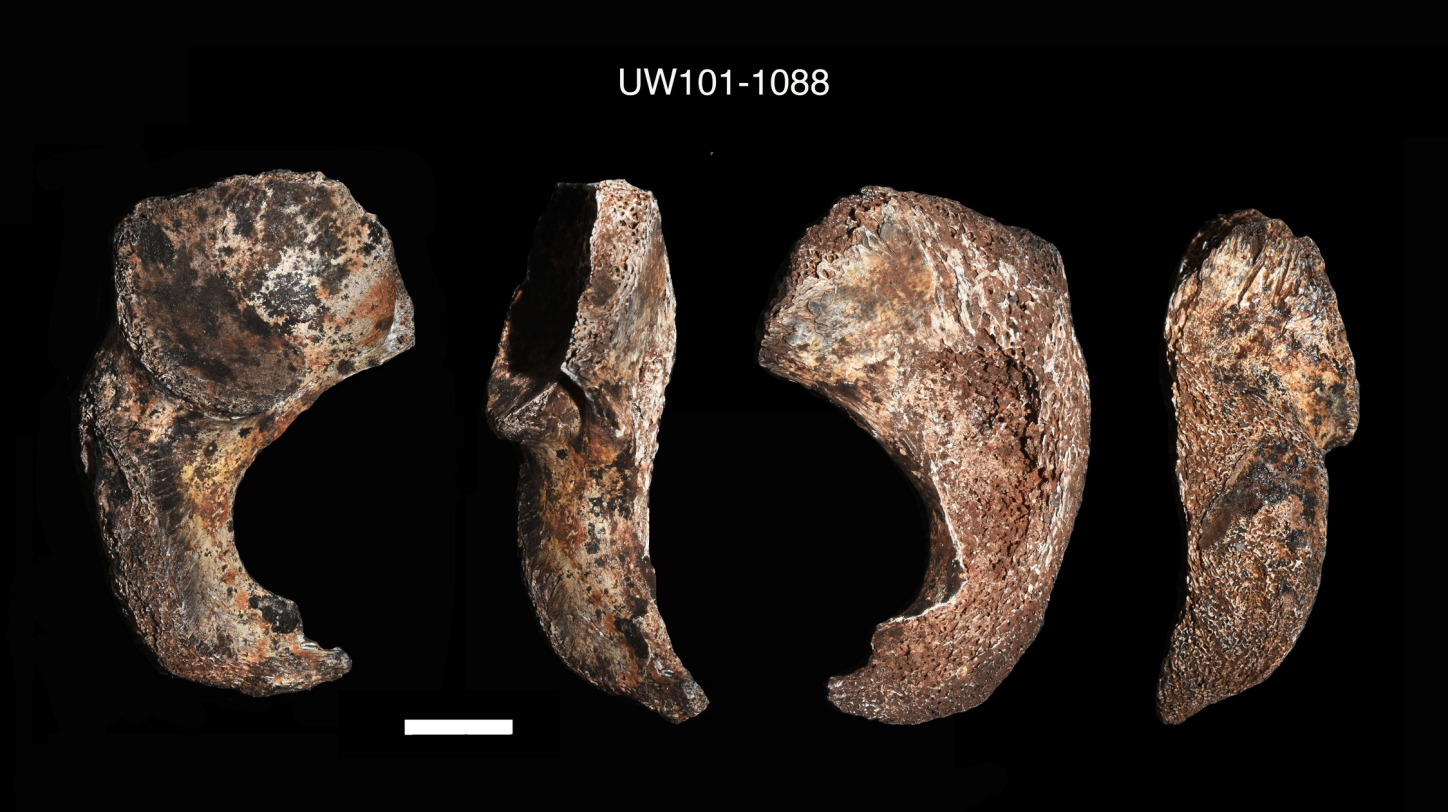
Right ischium (U.W. 101–1088).

**Fig 11:**
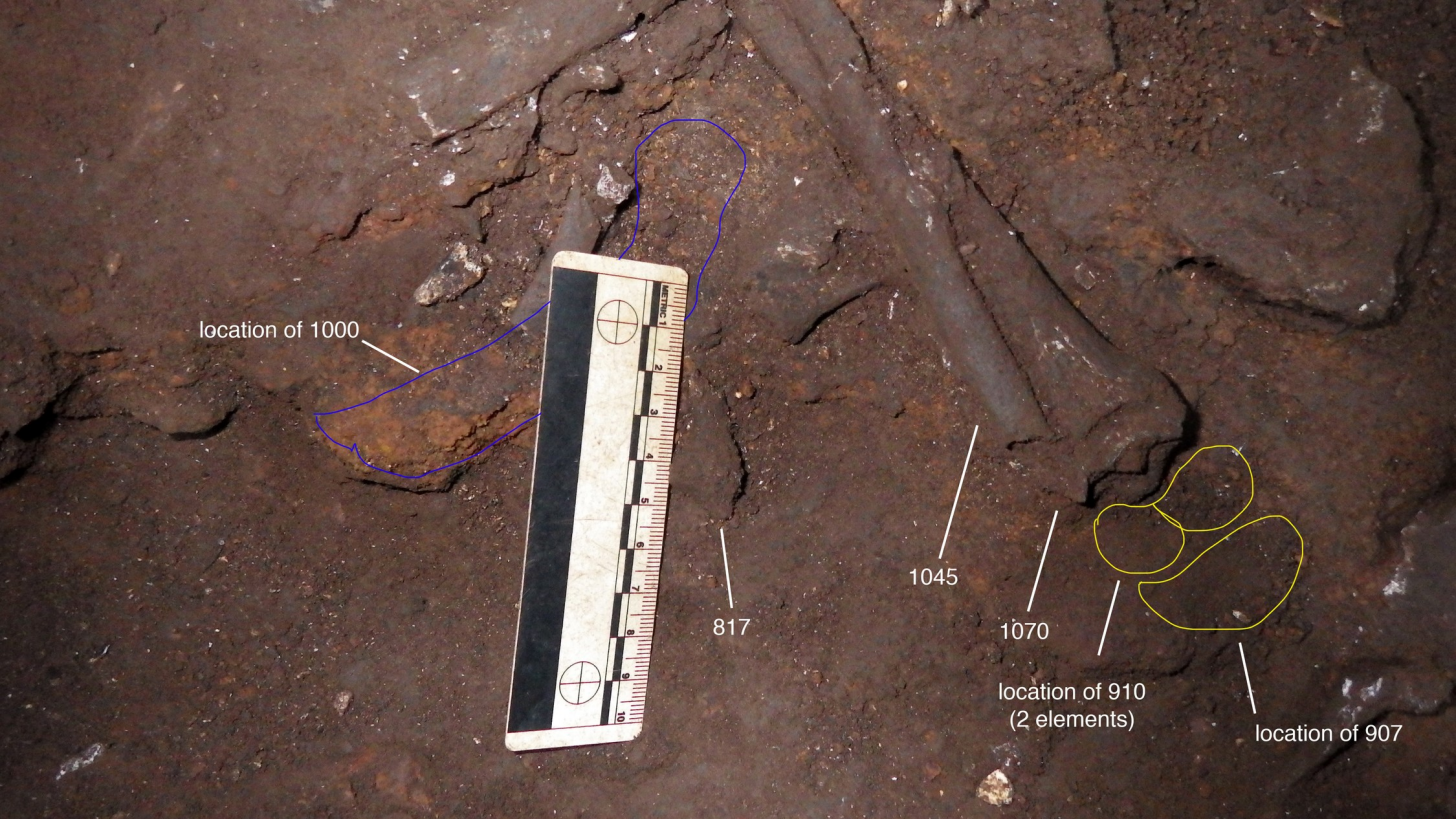
Excavation photograph. Image showing location of elements of right and left lower limb: U.W. 101–907, U.W. 101–910, U.W. 101–1000, U.W. 101–1070, U.W. 101–1045 and U.W. 101–817.

**Fig 12:**
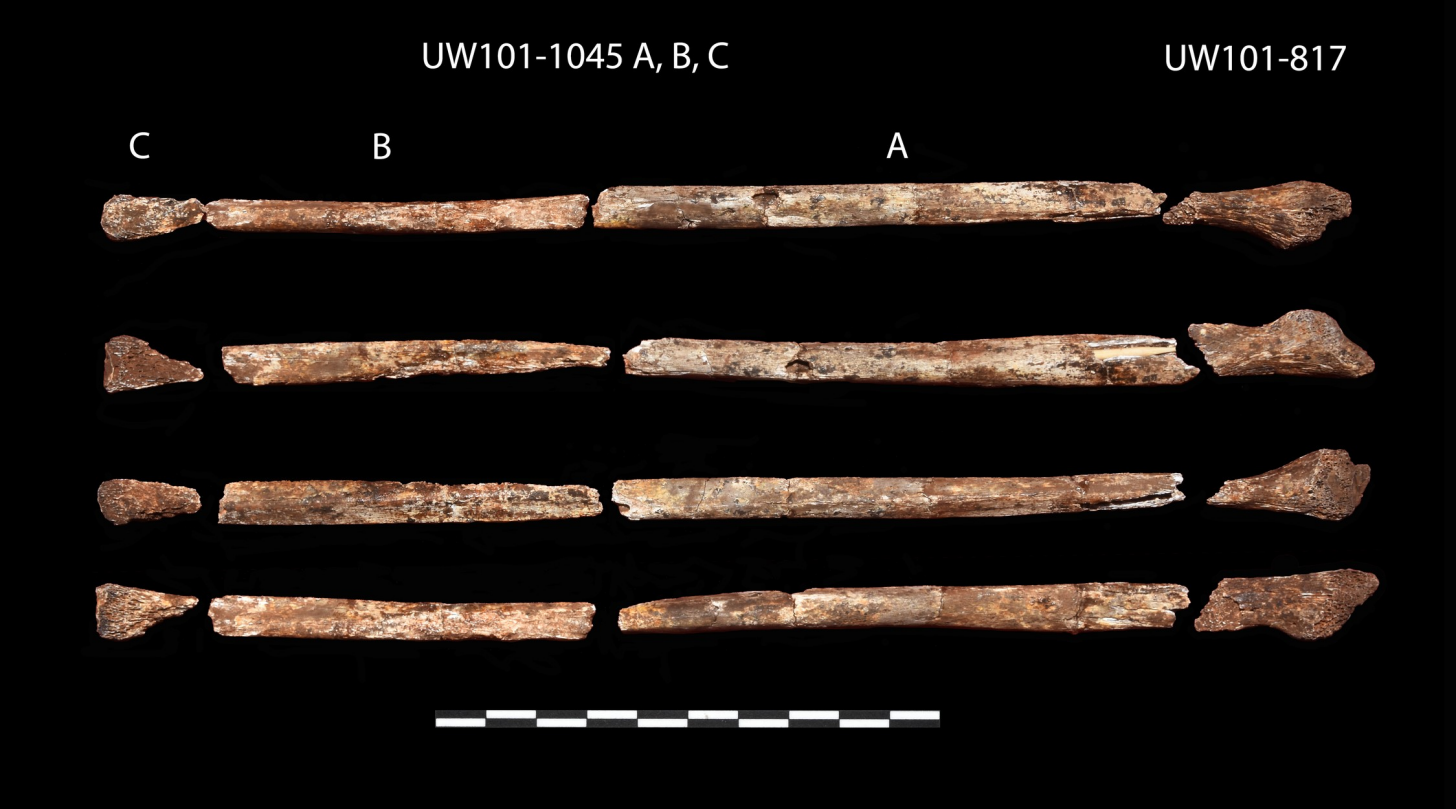
Left fibula, in 4 elements (U.W. 101–1045 a, b, c and U.W. 101–817).

**Fig 13:**
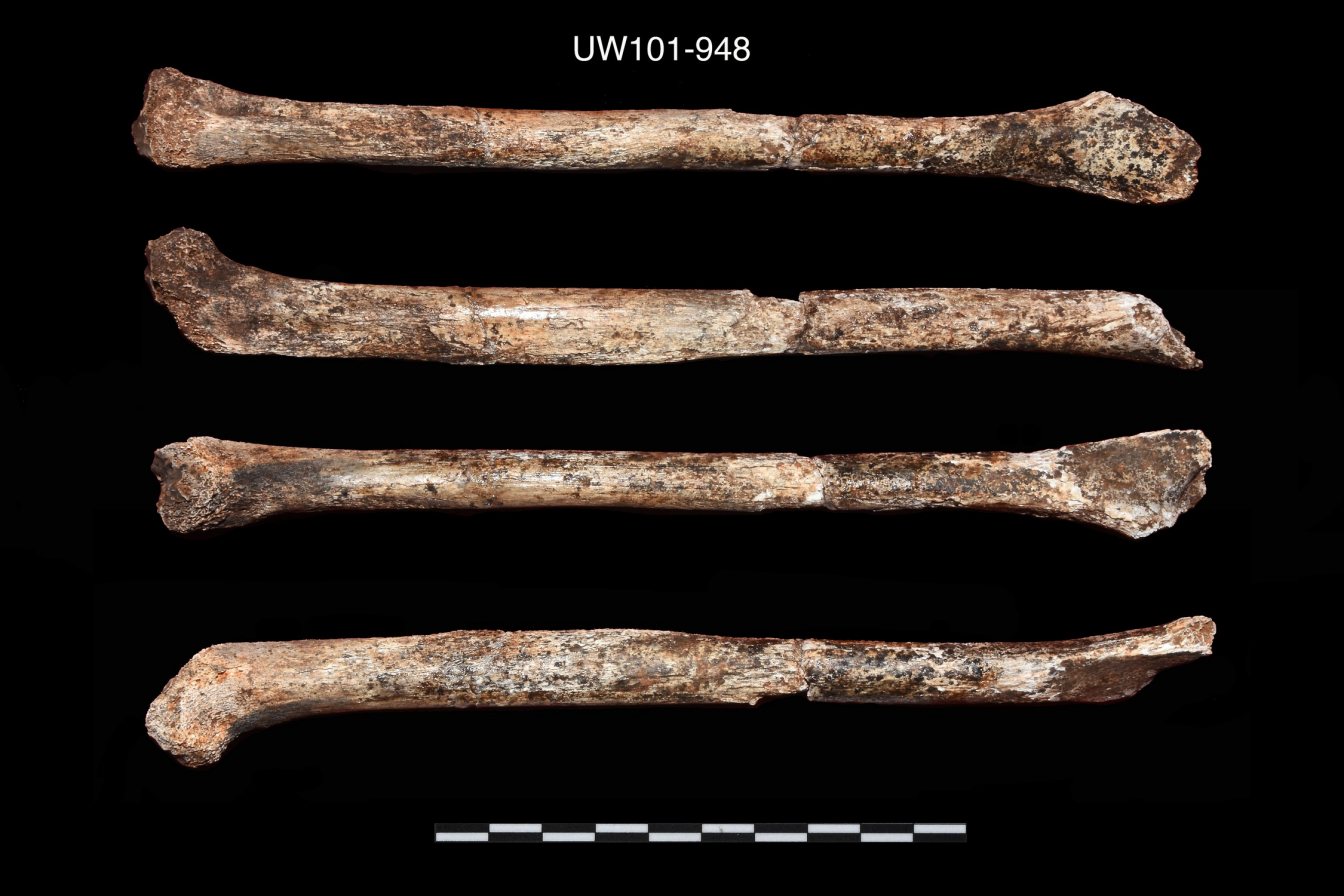
Right humerus, nearly complete. Composite of two elements, missing epiphyses (U.W. 101–948).

A right proximal femur fragment (U.W. 101–1000) was recovered from depth 1 (Fig 9A). The unfused head of this proximal femur fragment refits a right femoral head epiphysis (U.W. 101–1098), located at depth 2 (Fig 9B). A nearly complete right ischium (U.W. 101–1088) was found at the distal end of the articulated lower left limb in the excavation unit, at depth 2 (Fig 10). The distal segment of the left immature fibula (U.W. 101–817) was found adjacent to the articulated *in situ* ankle, at depth 2 (Figs 11 and 12). Two right humeral shaft pieces were found adjacent to the left tibia, and refit in the lab for a nearly complete humerus (U.W. 101–948) (Fig 13).

**Fig 14:**
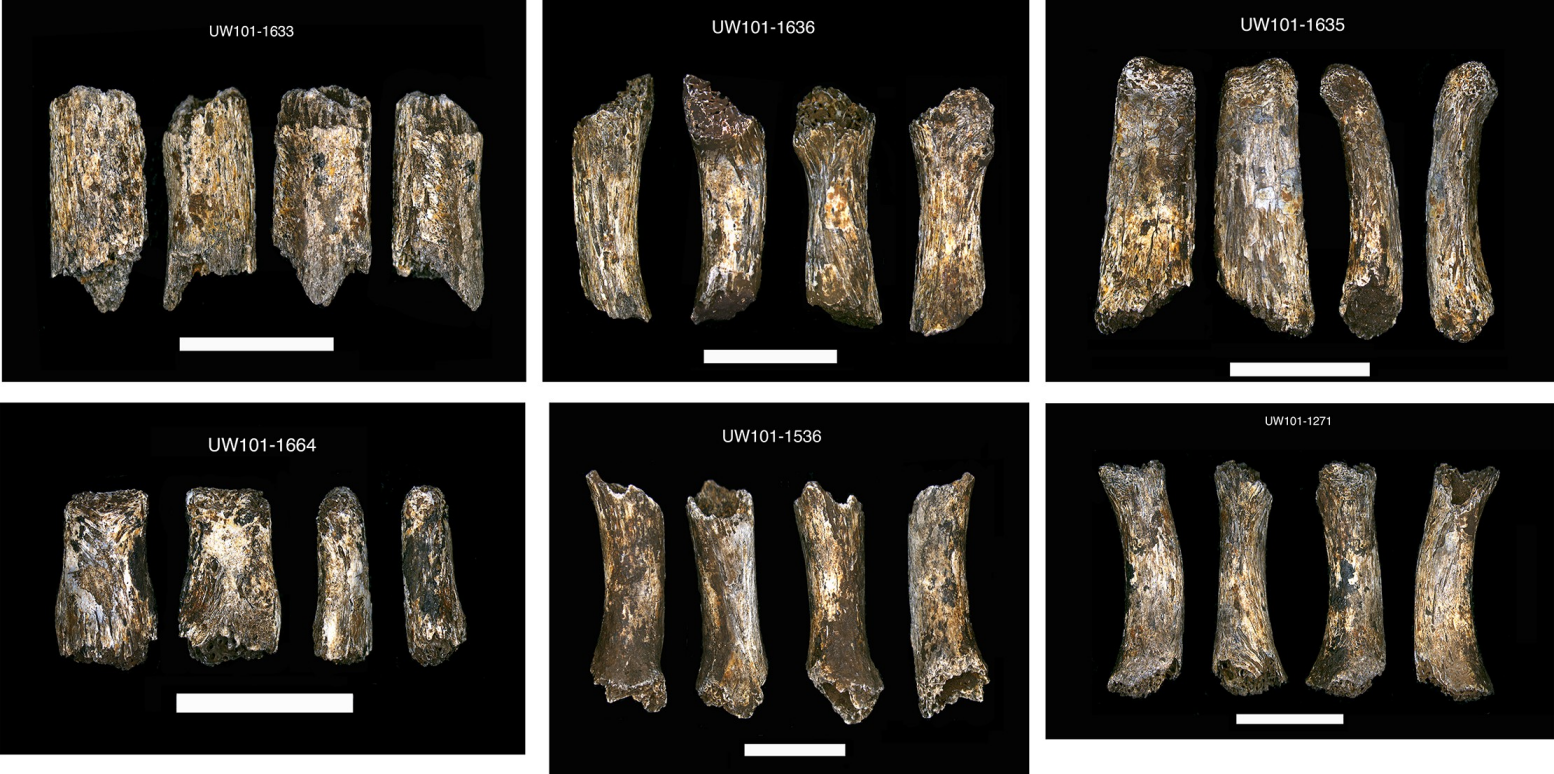
Hand remains found *en bloc*: a) unsided metacarpal shaft U.W. 101–1633; b) unsided metacarpal fragment; c) unsided proximal phalanx, distal shaft U.W. 101–1635; d) unsided intermediate phalanx fragement, U.W. 101–1664; and two additional age-consistent hand bones: e) metacarpal shaft fragment, unsided U.W. 101–1271; and f) metacarpal shaft fragment, unsided U.W. 101–1536.

Additional fragmentary hand bones were found near the articulated lower limb and hemi-mandible, and have provisionally been associated with DH7. Despite being small in size, these hand bones are consistent with each other in their developmental morphology and age indicators [17]. Two unsided immature metacarpal shaft fragments (U.W. 101–1633 and U.W. 101–1636), and two immature fragments from a proximal and intermediate phalanx (U.W. 101–1635 and U.W. 101–1664), provisionally attributed to “Hand 5” [17], were recovered *en bloc* at depth 2, and processed together in the lab (Fig 14A–14D). Two additional metacarpal shafts (U.W. 101–1536 and U.W. 101–1271), also attributed to “Hand 5”, were recovered from depth 2, and in spatial proximity to the partial mandible U.W. 101–377 (Fig 14E and 14F).

**Fig 15:**
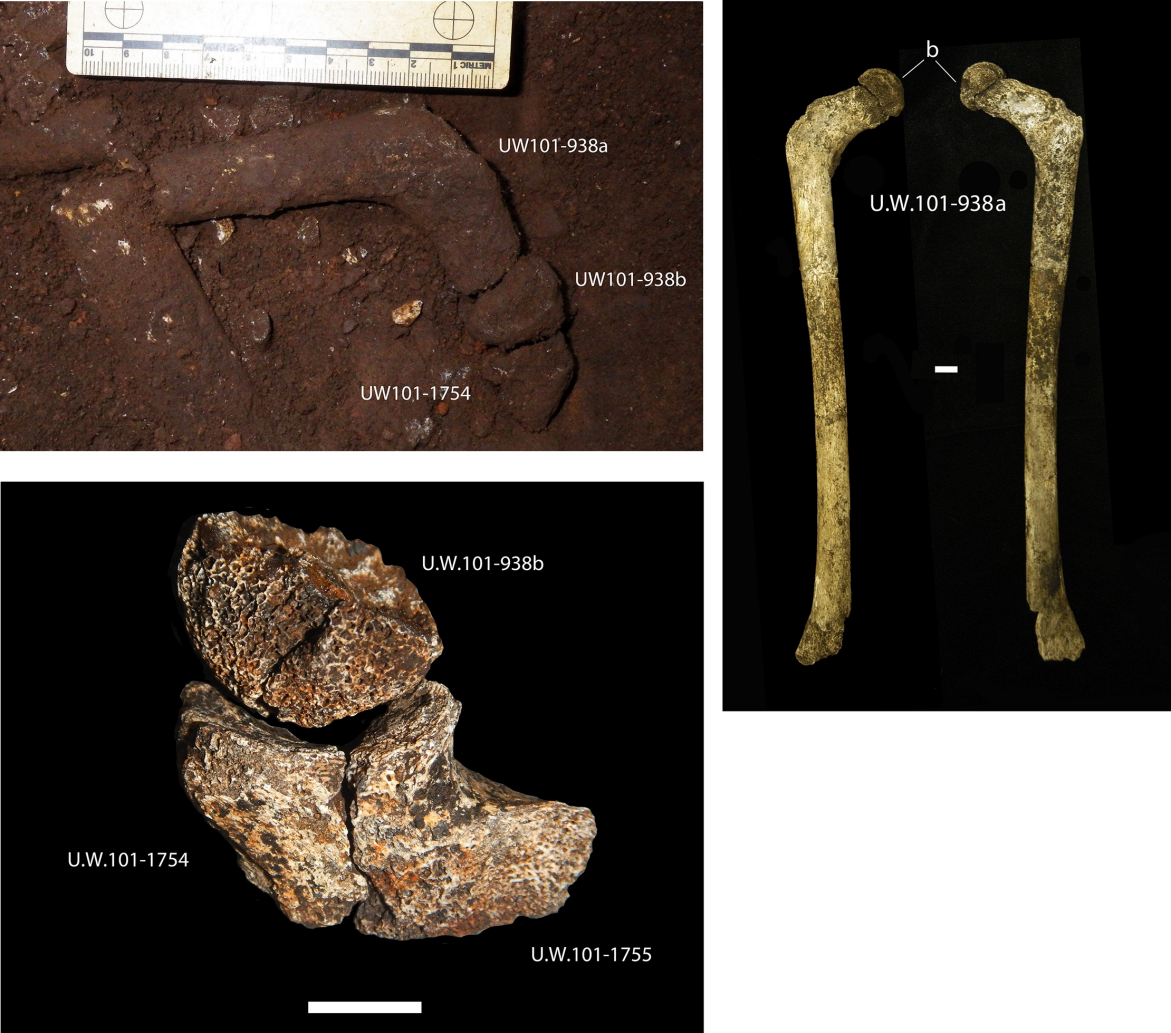
**Right femur (U.W. 101-938a, b) and pelvic fragments (U.W. 101–1754 and U.W. 101–1755).** a) *in situ* U.W. 101-938a, b and U.W. 101–1754; b) nearly complete femur from four refit pieces; c) articulation of U.W. 101-938b with U.W. 101–1754 and U.W. 101–1755.

Although dental MNI indicated only one ‘Late Juvenile’ [10,13], there is postcranial evidence of a second ‘Late Juvenile’ individual in the Dinaledi Chamber deposit [37]. This second ‘Late Juvenile’ is present in the northeastern portion of the excavation pit at depth 3, is not as complete as DH7, and duplicates elements of DH7. The material includes a right femur refit from four pieces (U.W. 101-938a), an epiphyseal head (U.W. 101-938b) and two right pelvic fragments (U.W. 101–1754 and U.W. 101–1755) (Fig 15). This partial lower limb orients towards the periphery of the unit, and the more fragmentary state may be a result of the position of the bones in the commingled pit. Bodies in the more compacted center of commingled deposits from forensic and archaeological mass graves undergo differential taphonomic factors, and may result in differential preservation from those on the outer edges [cf. 38].

Considering the entire fossil collection, we hypothesize that the ‘Late Juvenile’ material from the southwestern portion, and from depths 1–3 of the excavation pit, derive from the same individual as the associated postcranial material. We therefore provisionally attribute all this material to DH7. However, we note that the abundance of material in the present collection may still represent only a small fraction of the total amount of hominin skeletal material in the Dinaledi Chamber as a whole.

## Discussion

The conditions inside the Dinaledi Chamber have yielded an unprecedented number of *Homo naledi* fossils. In this case, the number of immature remains in the Dinaledi assemblage offers the possibility to expand our knowledge of stages of maturational processes in *H*. *naledi*. The identification of a partial older juvenile skeleton of *H*. *naledi* substantially adds to the database of associated immature partial skeletons in the human evolutionary fossil record.

The skeletal maturation of DH7 places it near the same maturational stage as partial skeletons of two other species, the MH1 holotype of *Au*. *sediba*, dated to 1.98 million years old [4,5,39], and the KNM-WT 15000 skeleton attributed to *H*. *erectus*, dated to 1.6 million years old [6,40–43]. All three partial skeletons share a combination of unfused long bone epiphyses and pelvic elements, indicating that growth was still occurring at the shoulder, hip, knee and ankle regions. Dentally, all three individuals lack erupted third molars, although the stage of premolar and canine eruptions varies across specimens. The age at death of KNM-WT 15000 is estimated between 8.3 to 8.8 years based on dental microhistology [42]; MH1 has been estimated to have been between 9–11 years old at death [5].

The DH7 partial skeleton (Fig 4) from the Late Middle Pleistocene contributes important new data to the field of hominin ontogeny. As designated, DH7 is consistent in skeletal maturity with MH1 and KNM-WT 15000, both of which exhibit an ape-like (non-human) maturational timing [5,29,41,42]. However, other body systems complicate the characterization of somatic maturity in DH7. Dentally, *H*. *naledi* appears to have a pattern of dental eruption which suggests an affinity with *H*. *sapiens*, with premolars fully emerged by the time the second molars are fully erupted; however, the pattern of dental root formation is more ape-like [44]. *H*. *naledi* has a unique dental pattern of surface enamel deposition unlike any other hominin, and unlike humans [45]. The mosaic of brain size and body size characters in *H*. *naledi* further complicates interpretations of the maturation pattern. At 480-610cc, *H*. *naledi* has an intermediate brain size between *Au*. *sediba* and *H*. *erectus* [12,46]. *H*. *naledi* has a stature more similar to *Au*. *sediba*, estimated at 143.5 cm for adults [5,46]. If *Au*. *sediba*, *H*. *erectus* and *H*. *naledi* shared a similar mode of maturity, this would suggest an age at death for DH7 between ~8–11 years old.

The phylogenetic arrangement of these three species relative to modern humans is uncertain [47,48]. The contrast in brain size among them is striking, raising the possibility of testing whether the maturation pattern in these immature skeletons relates to brain development. The maturational consistency between MH1 and KNM-WT 15000 has been used to support the hypothesis that Early Pleistocene hominins had not experienced the temporal shift in ontogeny that characterizes *H*. *sapiens*, irrespective of their brain or body size [5,29,43,49,50].

It remains possible that *H*. *naledi* had a slower maturation schedule than earlier hominins, similar to modern humans and Neanderthals. The species did exist contemporaneously with both early modern humans and Neanderthals in the Late Middle Pleistocene, and some evidence suggests that *H*. *naledi* may be a phylogenetic sister to these large-brained forms [47]. If so, the DH7 individual might be older than MH1 or KNM-WT 15000, ~11–15 years based on human maturational standards (Table 5). An intermediate pattern of somatic maturity is also possible.

**Table 5:** Estimated age in years of DH7 based on regions with skeletal maturity indicators, compared to *Homo sapiens*.

DH7: Bone elements (with catalogue numbers) and maturity indicators. See also [17,23,24,34,35,36].	*Homo sapiens* age
*Right humerus*, *nearly complete (U*.*W*. *101–948)*	
**Description:**	
A right humerus, no fusion of the proximal epiphysis. There is possibly a minute metaphyseal surface on the most lateral distal olecranon region.	≥11–13 female
	≥12–15 male
*H*. *sapiens*: The humeral head unites as early as 13 in females and 15 in males. The possibility of a metaphyseal surface on the distal region, in conjunction with the overall size, would suggest a younger age of 11–12 years [32].	
*Right proximal femur and conjoining femoral head (U*.*W*.*101-1000; U*.*W*. *101–1098)*	
**Description:**	
A proximal femur and partial femoral head that is unfused but mature in size and appearance.	≥12–13 female/male
*H*. *sapiens*: The femoral head unites between 14–19 years in modern humans, and although unfused, the mature appearance of the U.W. 101–1000 specimen suggests an upper age limit [32].	
*Right ischium (U*.*W*.*101-1088)*	
**Description:**	
The posterior acetabular epiphysis of this specimen is fully fused to form a continuous lunate surface. The acetabular surfaces and the ischial tuberosity epiphysis are unfused.	11–12 female/male
*H*. *sapiens*: Union of the posterior acetabular epiphysis begins between 10–11 years. The acetabula in modern humans begin fusing between 11–15 years in girls, and 14–17 years in boys. The ischial tuberosity initiates fusion between 13–16 years, leading to an estimated age at death of <13 years for U.W.101-1088 [36].	
*Right tibial shaft and left tibia with epiphyses (U*.*W*.*101-996; U*.*W*.*101-1070)*	
**Description:**	
For U.W.101-1070, the proximal epiphysis appears almost fully formed in size and shape, and is well-fitted to the diaphysis, although unfused. The distal epiphysis appears less well formed, but is also less well preserved.	≤11–14 female
	≤14–15 male
*H*. *sapiens*: The proximal tibial epiphysis initiates fusion as early as 13 years in females and 15 years in males. However, reported timings for the beginning of distal epiphysis fusion vary from as early as 11 years in girls and 14 years in boys [31], to as late as 14 years in girls and 15 years in boys [33].	
*Left calcaneus (U*.*W*.*101-907)*	
Description:	
This specimen retains a small region of metaphyseal surface on the posterior, medial aspect.	<12 female
	<15 male
*H*. *sapiens*: Fusion of the calcaneal epiphysis is advanced by 12 years in girls and 15 in boys. An unfused calcaneus, without the epiphysis for confirmation, is estimated to be a Stage IX or X [30] or roughly <12 years for females and <15 years for males.	

The late Middle Pleistocene age of *H*. *naledi* places the species as a possible contemporary in Southern Africa with our own species, *H*. *sapiens* (cf. [51]). *H*. *naledi*, with its unusual combination of primitive and derived traits, and its relatively recent existence, may shed light on the evolutionary adaptations that drove changes in the tempo of maturity, and possibly life history, in other late members of the *Homo* genus. The unprecedented assemblage of immature remains, plus the rare juvenile *H*. *naledi* partial skeleton, provide new opportunities to further the field of hominin ontogeny, and to explore the factors that shaped the evolution of extended maturity and life history in our own species.
